# A New Slicer-Based Method to Generate Infill Inspired by Sandwich-Patterns for Reduced Material Consumption

**DOI:** 10.3390/ma17225596

**Published:** 2024-11-15

**Authors:** Patrick Steck, Dominik Schuler, Christian Witzgall, Sandro Wartzack

**Affiliations:** Engineering Design, Friedrich-Alexander-Universität Erlangen-Nürnberg, 91058 Erlangen, Germany; steck@mfk.fau.de (P.S.); dominik.schuler@fau.de (D.S.); witzgall@mfk.fau.de (C.W.)

**Keywords:** infill pattern, additive manufacturing, lightweight design, load-corresponded design, function-integrated design, sustainable material consumption

## Abstract

This work presents a novel infill method for additive manufacturing, specifically designed to optimize material use and enhance stiffness in fused filament fabrication (FFF) parts through a geometry-aware, corrugated design inspired by sandwich structures. Unlike standard infill patterns, which typically employ uniform, space-filling grids that often disregard load-specific requirements, this method generates a cavity inside the component to be printed and fill the space between inner and outer contours with continuous, adaptable extrusion paths. This design enables consistent support and improved load distribution, making it particularly effective for parts under bending stresses, as it enhances structural resilience without requiring additional material. Simulations performed on a 10 cm^3^ test part using this method showed potential reductions in material consumption by up to 77% and a decrease in print time by 78%, while maintaining stiffness comparable to parts using conventional 100% grid infill. Additionally, simulations demonstrated that the new corrugated infill pattern provides near-isotropic stiffness, addressing the anisotropic limitations often seen in traditional infill designs that are sensitive to load orientation. This geometry-aware infill strategy thus contributes to balanced stiffness across complex geometries, enhancing reliability under mechanical loads. By integrating directly with slicer software, this approach simplifies advanced stiffness optimization without the necessity of finite element analysis-based topology optimization.

## 1. Introduction

Additive manufacturing (AM), commonly referred to as 3D-printing, has emerged as a transformative technology in recent years, gaining substantial relevance across various industries [1,2]. Its ability to create complex geometries and fast time-to-product are key benefits that differentiate AM from conventional manufacturing processes like CNC-machining, and enables the process for application in the high-tech sector and low-volume production [3]. However, besides the many advantages, additive manufacturing still has plenty of research potential. Fused filament fabrication (FFF), in particular, has experienced research hype in recent years, which has been driven first and foremost by the maker scene [4]. FFF is an additive manufacturing technique that generates objects in layers through the deposition of liquified thermoplastic polymers [5]. The layer geometry is formed by two main components: Firstly, the walls (often also referred to as the perimeter) which follow the outer contours of the model geometry. Secondly, the infill lies inside the remaining area of a layer and serves several functions. The infill geometry not only provides structural integrity, but also acts as a support for the above layers and thus ensures the printability of the part [6]. The individual layer geometry is generated using slicer software, which derives the tool paths for manufacturing based on model geometry and user input. Conventional infill geometries incorporate simple repeating patterns, such as squares or triangles [7]. These infill patterns are only space-filling and independent of the model geometry. The user can determine the density of the infill pattern by specifying a percentage value in the slicing software. As a consequence of the extrusion-based principle of FFF, the print time for a given part is determined by the volume of the required material. As the infill constitutes the majority of the volume of the parts, the infill geometry is a central factor in optimizing the print time, material consumption, and first and foremost—the stiffness behavior. These properties increase exponentially with increasing volume of the components, which is why there is great potential for optimization in this research field [8].

**Figure 1:**
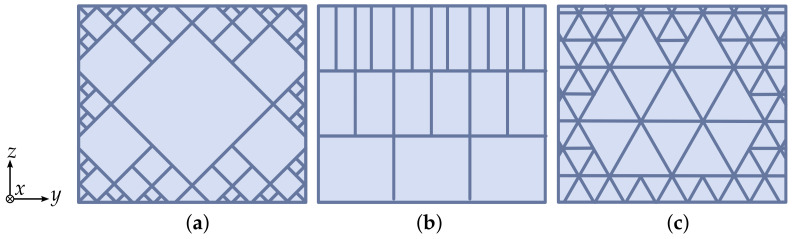
Different adaptive infill patterns with build direction in z-axis: (**a**) Rhombic cell; (**b**) Gradual; (**c**) Cubic-subdivision.

Various methods have been proposed to address this problem in the past. One approach is the possibility of generating geometries with variable density. Figure 1 shows a selection of such variable density infill patterns (rhombic cell, gradual and cubic-subdivision) according to Wu et al. [9].

The parts volume is subdivided into rhombic cells (see Figure 1a), whereby the size of the cells is increased towards the inside of the part using a density-weighted function. Increasing the density towards the boundaries ensures improved support capability for the outer surface. The gradual infill in Figure 1b uses conventional infill-geometry that is consistent across the layer, but varies the density incrementally along the z-axis. The cubic-subdivision infill in Figure 1c follows the same principle as the aforementioned rhombic-infill, but uses cubic triangle geometry (like quadrilateral pyramids). In contrast to conventional infill patterns, these adaptive patterns attempt to relocate the material towards areas of the layer where they are most effective. Most of them are already available in open-source slicer software, such as CURA Slicer [10]. In further studies, Wu et al. [8] developed a major stress trajectories method motivated by bone growth processes to generate stiffness-optimized infill patterns. Völkl et al. [11] developed a heuristic topology optimization algorithm for fiber-reinforced composites in which the outer shape of the structure is changed instead of the actual infill. The increase in stiffness is then realized by rotating the fibers in the direction of the maximum principal stress vectors. However, due to the necessity of a finite element analysis, these methods are difficult to implement for people without expertise in engineering. Furthermore, the functional integration and the focus on minimal compliance is a limitation. Yi et al. [12] used an approach that does not need any finite element analysis during the slicing process. An existing infill pattern is applied and the generated G-code is retrospectively related to the design proposal of the topology optimization software to generate a gradient-based infill. The advantage of this method is that the authors focus on tuning the extrusion width of the printed tracks and take the G-code as the processed data object. Hence, no finite element analysis (FEA) needs to be integrated into the slicing software. Another approach to reduce material consumption is the hollowing of a part geometry. A method presented by Wang et al. [13] utilizes a hollowing algorithm which divides an existing volume into a set of manufacturable cavities based on a printable overhang threshold. The method completely discards the infill geometry; the remaining shell is printed in solid material. Based on this method, Tricard et al. [14] propose an infill method that generates internal support ribs to ensure printability of the part while minimizing the material consumption. In this approach, the infill acts only as a support, whilst not contributing to the structural integrity of the part. As this method achieves a significant reduction in the required infill, the structural integrity of the part is only provided by the contours, like sandwich structures. A sandwich structure combines two load-bearing face layers with a low-density core that increases the structure thickness and, in consequence, provides an increased bending stiffness compared to a thinner structure of equal mass. Sandwich structures exist in a wide variety of materials and geometries [15]. Common materials for shell layers are composite materials or metal sheets. Common core materials are solid foam cores, hexagonal honeycombs and folded cores of various geometries [15]. Yazdani et al. [16] proved in their work that sandwich structures with alternating infill patterns can absorb more impact energy under bending loads in comparison to unidirectional infill patterns. The utilization of additive manufacturing techniques in sandwich design can be identified in various works. Peng et al. [17] experiment with FFF-printed bioinspired core structures. The authors developed a sandwich core design based on a triply periodic minimal surface, which also uses alternating patterns. The result was a higher stiffness than with conventional infill designs. Silva et al. [18] evaluated the mechanical properties of additively manufactured cellular cores with a graded structure. An increase in wall thickness to maintain part strength in large parts may result in longer manufacturing times and material consumption in comparison to an equivalent part with conventional infill. A periodic wave-like pattern conforms to the layer geometry and offers a consistent support between the outer and inner parts of the contours. However, due to the directional nature of the corrugated geometry, anisotropical mechanical properties are to be expected and offer further potential for improvement. The issue of the anisotropic mechanical properties of corrugated sandwich-cores has been thoroughly discussed in the literature [19,20,21,22,23]. Variations of the corrugated core geometry have been proposed to provide more isotropic properties. Seong et al. [22] tested a bi-directional corrugated core which is generated by extruding the cross-section in a zig-zag fashion, rather than in a straight line. A bending test of samples with different core orientations shows a quasi-isotropic bending response of the bi-directional corrugated core. A different variation of a bi-directional corrugated core is proposed by Ge et al. [23]. The authors create a novel core geometry inspired by corrugated sandwich cores. For better differentiation, this second variation will subsequently be referred to as “double-corrugated” geometry. Although the design of this double-corrugated core was manufactured with 3D-printing, the generation process of this geometry is not automated and needs a lot of time for designing by hand and integrating inside an own product. However, both the simulation and experiment conducted in [23] show nearly identical compressive properties for longitudinal and transversal core orientation. This shows clearly the potential that lies inside such lighweight pattern design. Bi et al. [24] formulate an approach for integrating such a corrugated inspired design guideline inside a slicing software. However, the authors used their approach for concrete printing and had, therefore, a different objective for application. Additionally, the pattern was limited to a 100% infill factor, with the aim to find a short way for a homogeneous filling without many travel paths. An approach which shows huge potential for infill patterns that incorporate stress is from Liu et al. [25]. The authors stated that for large structures, continuous paths have additionally advantages in comparison to established infill patterns. Furthermore the authors of [25] only investigated straight homogeneous patterns without changing the start and end points, which could have a further impact on the stiffness behavior of torsioned structures.

These approaches and methods highlight the need for straightforward, lightweight solutions to generate stiffness-optimized infill structures. In addition, only a few of them are prepared for integration into existing slicing software, which makes it very difficult to use them in the non-commercial sector. In the following chapters, an approach is presented whereby the core (infill) geometry is generated directly during the slicing process. This allows a user to benefit from the weight-saving properties of the sandwich structure without extensive modeling work. The method attempts to combine the approaches of hollowing and geometry-aware infill to achieve high structural integrity, reducing manufacturing times and material consumption.

This leads to the main research question, which will be addressed in the course of the present work:

*How can integrated methods in slicer software be used to modify infill algorithms in order to improve the stiffness, material consumption and manufacturing time of printed products?*

## 2. Materials and Methods

The proposed method is separated into two distinct steps, whereby the hollowing operation can be considered a pre-processing of the part geometry. The resulting geometry is then passed on to the slicer, where the layer paths including the specialized infill geometry are generated.

### 2.1. Hollowing Operation (Pre-Processing)

The hollowing operation is used to obtain a modified part geometry resembling a shell of the original geometry with constant thickness. The hollowing operation is executed independently of the slicing operation, allowing the user to run the software including the pre-processing step, or alternatively to import geometry that has already been hollowed in CAD or using other methods [26]. The open-source software Blender v2.93 was used to achieve the hollowing operation, using its “solidify” modifier. The operation is executed in three steps:

1. Import of part geometry (STL mesh)
2. Hollowing of part geometry (thickness is set by user)
3. Export of part geometry (STL mesh)

Blender provides a Python interface, which allows for the handling of geometry within a Python code that passes the resulting geometry on to the slicer.

### 2.2. Path Planning

**Figure 2:**
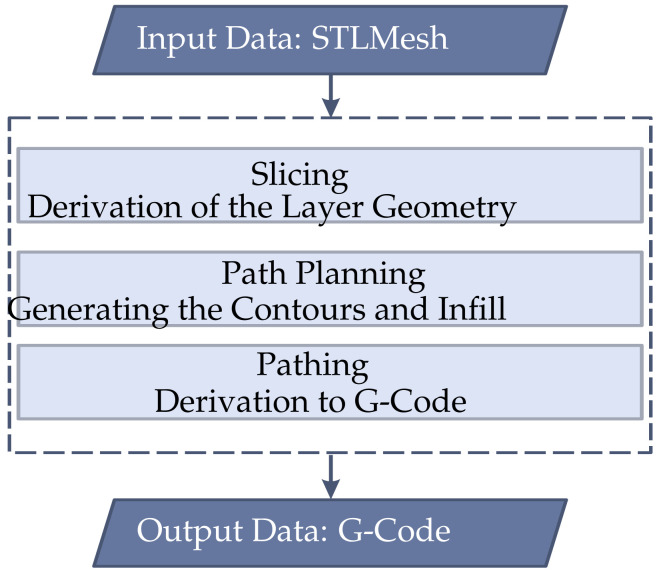
General process steps for generating G-codes. The input is an STL mesh. This mesh is then cut up. Paths are then planned from the individual slices by meshing again. The paths are then generated and finally output as G-code.

Path planning is a key step in the 3D-printing process that enables the efficient conversion of digital models into physical objects. The process begins with the provision of input data, typically in the form of an STL mesh file, which describes the geometry of the object to be printed (see Figure 2).

The model is then broken down into layers (slicing) to derive the geometry of each layer. The contours and the infill, i.e., the internal structures, are then generated in the actual path planning. Finally, the path is derived to create the G-code, which contains the machine instructions for the printer [27].

**Figure 3:**
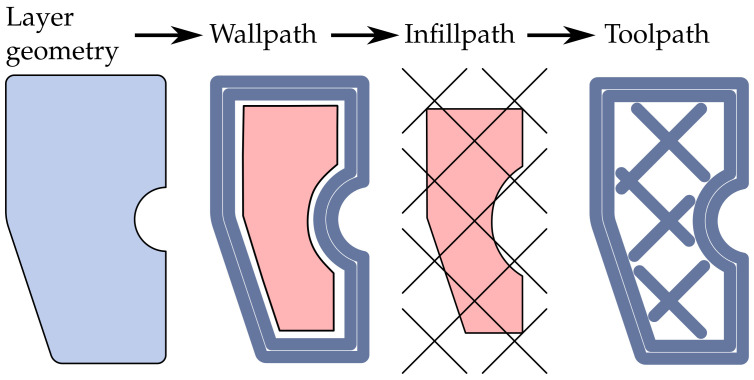
Tool path generation process: This starts with the layer geometry. The wall paths are then defined. Next, an infill pattern is placed over the remaining inner geometry and, finally, the tool paths are generated.

The tool path generation process begins with layer geometry, where the 3D model is sliced into horizontal layers. Next, the wall path is generated, outlining the external contours of each layer, which defines the object’s surface quality and precision. Following this, the infill path is created within the walls to provide internal structure, balancing strength and material usage. Finally, the complete tool path is generated, combining the wall and infill paths into a G-code file that the 3D-printer follows to build the object layer-by-layer. Figure 3 shows the described tool path generation process.

The FFF user has access to a wide variety of different slicer software, including both closed- and open-source options. Mandoline Slicer [28] is an open-source Python-based slicer that is limited to only the basic functionality required to generate FFF tool paths from an imported mesh. The code relies on an open-source library Klipper [29] for geometry handling, which stores 2D polygons as lists containing their vertices. Klipper provides a toolbox including offset and Boolean operations used to modify geometry. Due to the concise nature of its code, Mandoline was chosen in this work as the basis for the implementation of the new infill algorithm.

Mandoline Slicer generates the layer paths in three steps. The first step of the slicing process gives the process its name and is used to derive the layer geometry of the individual layers for manufacturing. This is achieved by calculating the intersection of the STL-mesh with a plane at a set z-value [26]. The resulting geometry is a 2-dimensional area representing the solid portion of the part. In a second step, the contour paths are derived from the layer geometry. An offset is applied to the outer contours of the layer geometry, corresponding to the extrusion width. The offset operation is repeated depending on the number of contour extrusions set by the user. Infill paths are then generated in the last step by intersecting an infill pattern with the remaining area (red). Once the layer paths are finalized, the geometry is used in the path-planning operation to generate a machine-readable G-code for manufacturing. The infill algorithm proposed in this paper supplements the final step of geometry derivation and is described in Section 2.3.

### 2.3. Method for Corrugated Infill Pattern

The use of the hollowing operation and subsequent slicing results in a part with an outer and inner shell, which are connected by the infill. As such, the cross-section of the part resembles that of a sandwich structure. Whilst a reduction in print time and material consumption can be achieved through the use of conventional infill geometry, potential for improvement can be identified in several aspects. As the infill geometries orientation remains constant throughout the part, its orientation relative to the parts surface is dependent on part geometry. This can lead to inhomogeneous mechanical properties throughout the part. Furthermore, the resulting infill geometry consists of a large number of independent disconnected segments, which in consequence is suboptimal for 3D-printing. The short extrusion lengths require great precision in the extrusion system, and can result in blobs or under-extrusion. The many accelerations during these short segments as well as the positioning moves required, increase print time and can induce oscillations into the printer.

The proposed infill geometry resembles a corrugated sandwich core, which alternates between two face layers. The continuous geometry allows for the infill to be printed in one continuous extrusion. Additionally the geometry-aware nature of the infill allows for a more consistent support of the inner and outer shell throughout the part. In order to successfully generate such an infill pattern for a layer, two requirements are set regarding the layer geometry:

1. The geometry requires two closed contours that are clearly assignable to each other; therefore, the corrugated pattern can alternate between them.
2. An equidistant space between the contours is required to ensure the consistency of the infill geometry.

**Figure 4:**
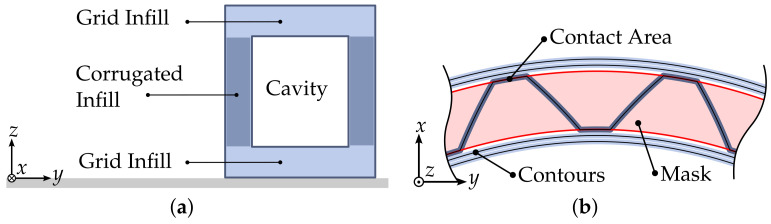
(**a**) Placement of hollowing-based infill in layers including a cavity. (**b**) Placement of corrugated infill pattern between inner and outer contours.

A distinction of cases is conducted at the beginning of the infill method which tests the requirements stated above. If the requirements are fulfilled, the corrugated infill pattern is generated, otherwise a conventional infill pattern is used. The infill method is executed consecutively after the contour method. The area in which the infill is to be generated is defined by the innermost wall paths, with an offset applied to account for the extrusion width. The resulting area is referred to as a mask (see Figure 4) and is subsequently used to derive the infill pattern.

The simplest form of a corrugated core pattern is a triangular pattern that alternates between points on two contours. In order to improve the bond between the infill and contour paths the contact points are extended to form contact surfaces, resulting in a trapezoidal corrugated pattern. In principle, the infill pattern follows the mathematical behavior of a periodic sigmoid function, such as Equation (1).

(1) $$f\left(x\right)=A\left[\frac{1}{2}+\frac{1}{2}tanh\left(Bsin\left(Cx\right)\right)\right]$$

**Figure 5:**
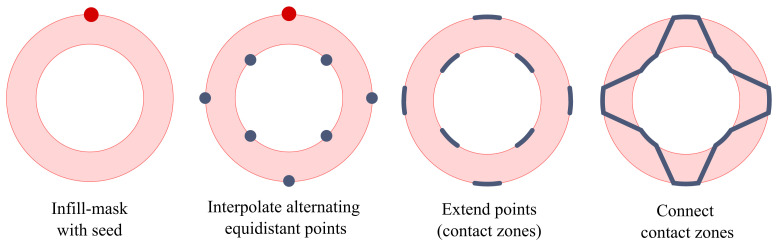
Graphical explanation of the corrugated infill algorithm. Red dots mark the initial seed points. Blue dots mark the contact points with the outer and inner shell. Blue lines mark the contact regions (paths) with the outer and inner shell.

This function has a periodic sigmoid-like form. The constant *A* controls the amplitude, determining how large the output will be. The tanh function ensures smooth transitions between high and low values, modulated by the sine wave sin(Cx), which adds periodic oscillations. *B* influences how steep or smooth the transitions are, while *C* controls the frequency of these oscillations. This setup allows for a periodic yet smooth function that oscillates between values in a controlled manner. The contour segments that form the interfaces are derived in two steps. In a first step, the contours are scanned starting from a seed point to interpolate equidistantly spaced points according to the number of interfaces specified by the user. The example displayed in Figure 5 shows a corrugated infill pattern with the number of interfaces set to eight.

Accordingly, the first point is placed at 0% from the seed, the second point at 12.5%, the third point at 25% and so on. As the algorithm alternates between the outer and inner contour, an even number of interfaces is required. In a second step, the contact points are extended to form the interfaces. This is achieved by interpolating new points before and after each contact point that form the start and end of the interface. The interface consists of the start point, end point and the contour points between the two. The length of the interface is set by the user as a percentage of the circumference. The infill geometry is completed by linking the interfaces into one continuous path. The segments connecting the interfaces form the core structure that supports the outer and inner contour against each other. As the infill geometry is individual to each layer, actions have to be taken to enable the alignment of the individual layers, and as such, the formation of a cohesive infill structure throughout the part. The alignment between layers is achieved by passing on the previous layers seed coordinates to the new layer. The new layers seed is then oriented above the previous seed.

The infill function is called as part of the tool path generation process, as described in Section 2.3. Required inputs for the method are the layer geometry consisting of an inner and outer contour, the interface count and the contact size. Optionally a seed coordinate from a previous layer can be used. In the first step, the new seeds are determined. A function is called to find the points on the inner and outer contour that lie closest to the previous seeds. If no seeds are provided, a random seed location is chosen. In line 10, a loop is initiated for the calculation of the individual contact interfaces. The algorithm alternates between the inner and outer contour. Each interface consists of points along the layers inner or outer contour. To determine these points, a function P on Contour was implemented. This method is used to calculate a point on a contour that lies a given distance away from another point (seed) on the contour. The distance between the seed and the returned point is inputted as a fraction of the total circumference. The method passes through the points of the contour until the distance between the points passed exceeds the requested distance goal. When this is the case, the searched point is known to lay on the last line segment. A linear interpolation is used to calculate the point. The P on Contour is used to determine the start and end point of each interface. The start and end points are used to extract the included points that form the interface. In a final step, the points are joined to form the infill geometry. As the algorithm follows the circumference, the points are in order and can be used directly for a continuous tool path. The implementation of the method as pseudo-code is shown in Algorithm 1.

**Figure 6:**
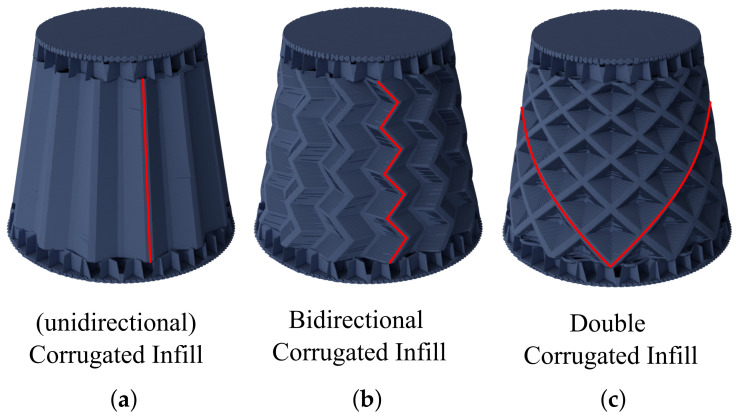
Variation in the corrugated infill pattern by alternation of the seed path (red lines): (**a**) Unidirectional corrugated infill, in which the seeds are always placed collinear. (**b**) Bi-directional corrugated infill, in which the seeds are positioned alternately at a predefined distance. (**c**) Double-corrugated infill, in which the seeds are distributed at a constant distance from a starting point in both directions.

Both of the described bi-directional variants (see Section 2) of the corrugated geometry were implemented into the corrugated infill algorithm. As described previously, a seed is used to pass the orientation of a previous layer’s infill onto the current layer. Figure 6 shows three infill types with the corresponding seed-path marked in red.
**Algorithm 1** Corrugated infill pattern algorithm. 1: Initialize the preferred type of infill pattern and calculate the position of anchor points (seeds) 2: l,i,c,o←0▹ layer, interfaces, contact, old seeds 3: **if** infill type is corrugated **then** 4:   infill, seeds ←corrugated infill(l,i,c,o)) 5: **end if**
 6: **function** corrugated infill(l,i,c,o) 7:   s←find closest points(l,o)▹ seeds 8:   p←[0,0,0]▹ points 9:   **while** n<i **do**10:     r←n/*i*▹ ratio11:     **if** *c* is even **then**▹ inside contour12:       b←P on contour(l.in,s.in,r-c/2)13:       e←P on contour(l.in,s.in,r+c/2)14:       t←points between(b,e)15:     **else**▹ outside contour16:       b←P on contour(l.out,s.out,r-c/2)17:       e←P on contour(l.out,s.out,r+c/2)18:       t←points between(b,e)19:     **end if**20:     n←n+121:     p←p+[b,e,t]22:   **end while**23:   f←create line segments(*p*)24: **return**
  *f*25: **end function**
26: **function** P on contour(k,s,r)27:   k←reorder(k,s)28:   g←circumference(*k*)+*r*29:   m,d←0▹ segment, distance30:   **while** h<g **do**31:     h←distance(k[m],k[m+1])▹ length32:     d←d+h33:     m←m+134:   **end while**35:   w←g–*d*36:   u←w/*h*37:   p←Interpolate(k[m],k[m–1],u)38: **return** 
*p*39: **end function**

To achieve the wanted geometry, the infill method was extended to incorporate an offset variable. The offset is used to shift the seed point, and in consequence, the resulting infill pattern, along the contours of the mask. The offset can, thus, be used to manipulate the direction the infill geometry propagates through the part. The bi-directional corrugated infill is achieved by alternating between positive and negative offsets. The double-corrugated infill (the right picture of Figure 6) is achieved by executing the infill algorithm twice, once with a positive and once with a negative offset. The offset is set to equal the layer height, resulting in a direction of roughly 45° for each component.

## 3. Results

The results of the paper are presented below. A new infill pattern is shown, which is based on the previous information in Section 2 on corrugation patterns and hollow structures. First, the printing time and material consumption of various infill patterns (standard and the new ones in this article) are shown using a simple vase example. This example is also used to demonstrate the versatility with regard to discontinuous outer contours. Afterwards, the stiffness of a tube with a constant print volume is compared using an FEA simulation.

### 3.1. Simulations

The performance of the novel infill pattern is investigated with regards to the objectives stated in the research question. Firstly, the reduction in print-time and material consumption is tested for parts of varying dimensions. Secondly, an FEA Simulation is conducted to evaluate the bending stiffness of the different infill geometries. Finally, a simulative proof shows the aforementioned thesis of higher stiffness with constant volume for different infill patterns.

#### 3.1.1. Print Time and Material Consumption

**Figure 7:**
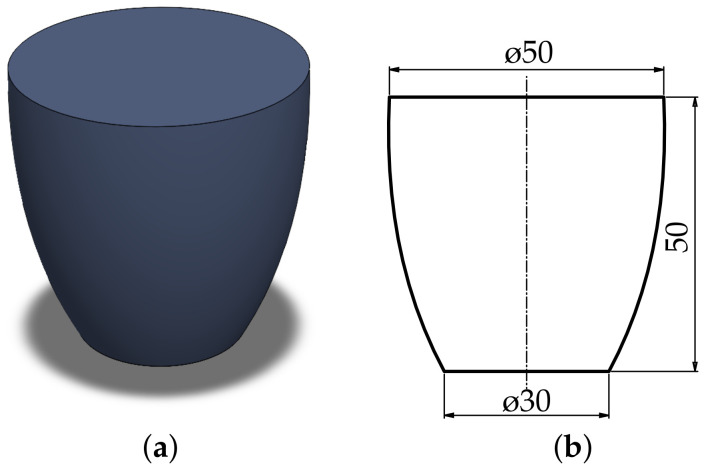
Test part geometry: (**a**) Isometric view. (**b**) Outer dimensions of the test component for comparing the different infill types. (All units in the figure are in mm).

In order to assess the potential for reduction in material consumption and print time, a test part was sliced with different infill settings. As discussed previously, the impact of the infill increases with rising part size. Accordingly, data were collected for parts of multiple sizes through repeated upscaling of the test part geometry displayed in Figure 7.

In order to ascertain benchmark values, the parts were sliced conventionally with a 25% grid infill. Afterwards, the hollowing operation described in Section 2.1 is applied and the part is then sliced with grid infill and the novel corrugated infill geometries documented in Section 2.3. For comparison, the geometry-related slicing settings are listed in Table 1.

**Figure 8:**
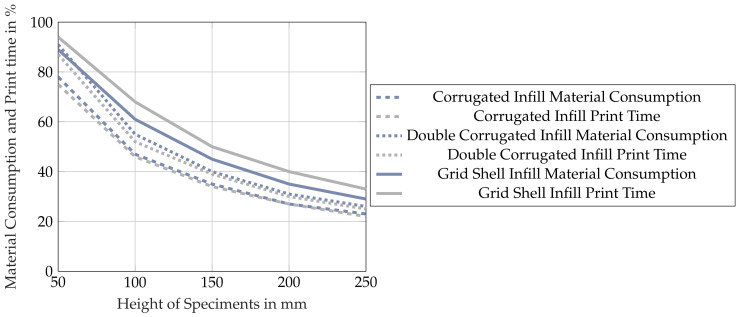
The behavior of print time and material consumption with increasing geometry height for the different infill types using the sample geometry from Figure 7. The material volumes were generated simulatively using the software Klipper v0.11 (see Section 2.2).

The print time and material consumption are shown in Figure 8 for part heights of 50–250 mm.

The values are normalized relative to the grid infill, which is used as a benchmark. The application of the hollowing operation achieves a significant reduction in material consumption across all part sizes. As predicted, the material savings increase with the part size and show a reduction from 11% to 71%. Further improvement can be seen when utilizing the corrugated infill structures discussed in Section 2.3. The corrugated infill structure achieves the lowest material consumption whilst the double-corrugated variant lies in between the grid infill and the corrugated infill. The print time follows a similar pattern as the material consumption, and the performance of the different infill types is ranked in the same order. This behavior is consistent with the proportional relationship between the part volume and the print time that has been discussed in Section 1. However, it is noticeable that the reduction in print time for the corrugated infill types is disproportionally large in comparison to the reduction in material. For example, for the 200 mm height part, the corrugated infill achieves an 8% reduction in material consumption compared to the grid infill, whereas the print time is reduced by 13%.

#### 3.1.2. Three-Point Bending Simulation

**Figure 9:**
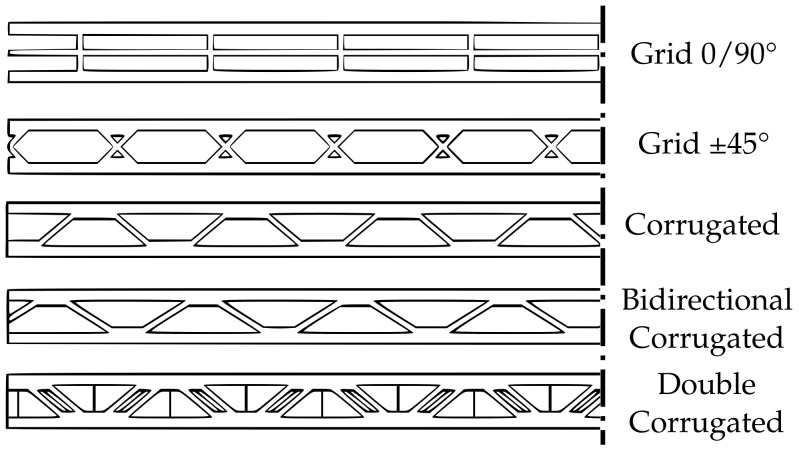
Geometry of the different three-point bending specimens. The geometries are simplified in the middle as symmetric.

To evaluate the bending stiffness of the novel infill structures, an FEA was conducted. As all infill geometries are directional in nature, a three-point bending test was simulated with the infill geometry oriented both longitudinally and transversely to study the influence of the infill orientation on bending stiffness. The sample geometries were designed in CAD and resemble panels with a side length of 100 mm and a thickness of 5 mm. The parameters used to define the infill geometry vary between the infill types; accordingly, a direct comparison is difficult to implement. As material consumption and specific stiffness are relevant properties, the geometries were adapted so that all samples have near identical volume (mass) (see Figure 9).

**Figure 10:**
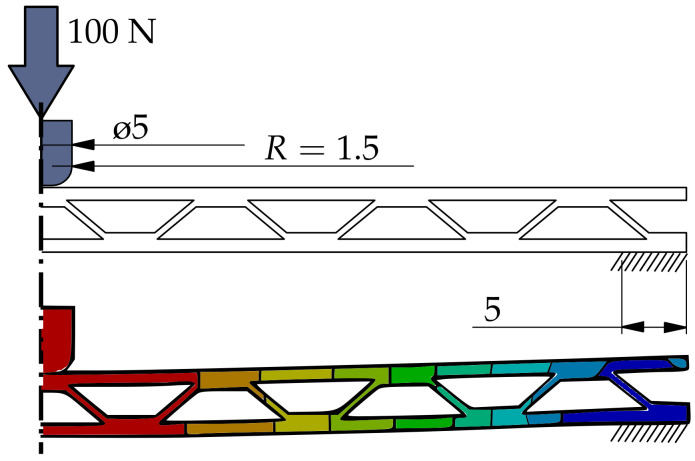
Qualitative deformation by three-point bending test using the corrugated infill as an example. The example is simplified in the middle as symmetric. The whole length of a specimen is 100 mm and the thickness is 5 mm. (Dimensions are in mm).

As the double-corrugated infill geometry is, in principle, two overlaid corrugated infill structures, it is naturally denser and could not be adjusted to the volume of the other samples. The volume and mass of the double-corrugated sample is, thus, 15% higher than the other samples tested. The grid infill was tested in two orientations to show the influence of the infill orientation within the part. The simulation is setup in the software ANSYS 2023 R2. The boundary conditions are displayed in Figure 10.

**Figure 11:**
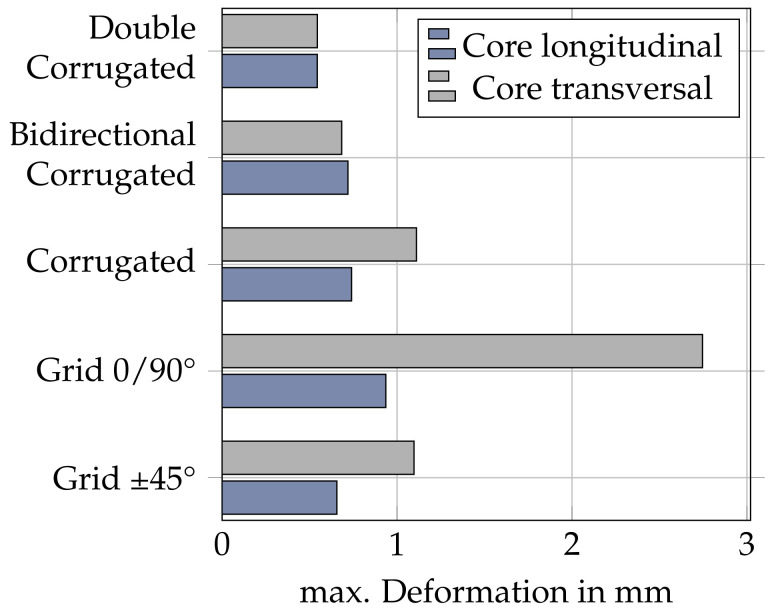
Maximum deformation of simulative three-point bending test specimens. Gray are bending values for the infill patterns which are along the bending line, and blue are transverse to the bending line. The deformations were simulated using Ansys 2023 R2 software.

In the real printed part, the anisotropic material properties of the extruded material strands and the inter-layer bonds result in a more complex deformation behavior. For this purpose, a three-point bending simulation of the different infill patterns and the different bending directions was carried out and the maximum deformations were compared. Figure 11 graphically shows the different maximum deformations.

For the simulation, an isotropic material model was used for simplification and to isolate the effect of the infill geometry. The bending behavior of the ±45° grid infill is shown to be anisotropic, with the transverse core orientation performing significantly worse than the longitudinal orientation. The effect is amplified for the 0/90° grid infill for which the maximum deformation differs by a factor of three, depending on the core orientation. The corrugated infill mirrors the ±45° grid infill in its bending performance. The bi-directional corrugated infill shows near isotropic behavior. It is the only one of the tested geometries where the deformation of the transversal core orientation is lower than the longitudinal orientation. The double-corrugated infill shows the lowest deformation and identical bending response for both orientations tested.

#### 3.1.3. Stiffness and Stress Comparison with Constant Volume

**Figure 12:**
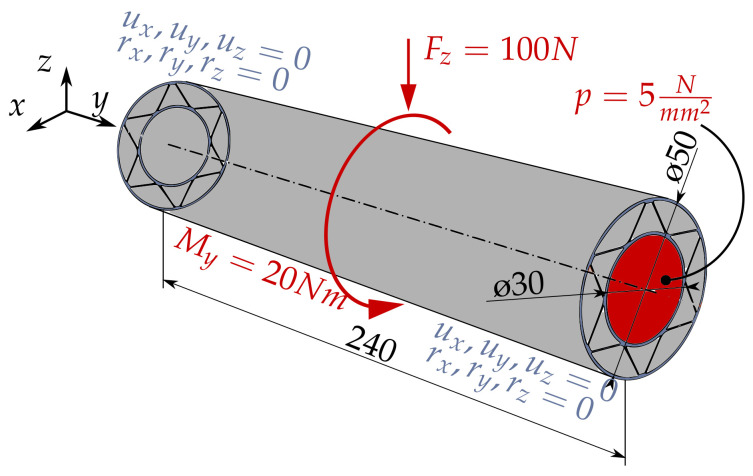
Three-dimensional sketch of the test part for stiffness and stress comparison: force $F_{z}$ and moment $M_{y}$ are aligned in the middle of the structure, *p* is a pressure that occurs in the inside of the pipe. The pipe is fixed through both end surfaces in the *y*-direction. (All units in the figure are in mm).

After the deformation of a representative infill fragment was investigated, the stiffness and the stresses occurring could be analyzed on a realistic application example for various load cases. In Figure 12, the used setting can be seen as an example for the corrugated infill.

The test part is a rotation-symmetrical pipe in which the cross-sectional area was left the same for all infill types in order to generate a constant volume with a constant length. This ultimately leads to valid comparability of the results. The outer and inner perimeters basically consist of two paths and are, therefore, 0.8 mm in width. However, as an attempt must be made to keep the area or volume constant, these are assumed to be variable. The infill is made out of one path for all infill types, which is why all thicknesses for all infill types are only 0.4 mm thick. The material used was amorphous PET (density 1339 kg/m^3^; Poisson’s ratio 0.3887) with a Young’s modulus of 2898 MPa, a shear modulus of 1043 MPa and a bulk modulus of 4339 MPa. A total of three different infill types (grid, corrugated, bi-directional corrugated) were examined, each with three different load cases. Common to all load cases is the fixed constraint on the two outer surfaces in the y-direction. In the first load case, a point load of 100 N acts in the center of the component in the negative z-direction. In the second load case, a moment of 20 Nm acts on the outer surface of the tube. The third load case is a static pressure of 5 MPa inside the pipe.

The element type used was SOLID186 with an element size of 1 mm. In addition, the sweep method was applied to the outer cylinder in order to obtain a uniform mesh with quads. Furthermore, a mesh refinement method was applied to the contact area between the infill and the outer contour. Since these are static load cases, an implicit solver (preconditioned conjugated gradient (PCG)) was used. The loads were applied directly in one time step.

In Table 2, the maximum deformations and stresses for the described load cases and infill types at constant volume are compared.

The color distributions of the individual analysis results are normalized to the maximum values of the infill with the highest deformations/stresses (in all cases to the grid infill). After applying the first load case, the greatest stresses were found in the cross areas of the grid infill. In addition, high stresses can be seen in the outer areas (z− and z+). The high inhomogenity of the stress distribution is remarkable, which indicates a bad utilization of the available material. This does not happen with the corrugated infills. Here, the stresses are homogeneously distributed across the infill, which are arranged like a truss. This leads to a uniform resistance and, finally, results in a lower deformation (0.0914 mm) against bending compared to the grid infill (0.1201 mm). This becomes more evident with the stresses occurring during torsional loads. As the infill opposes the shear during torsion, the infill almost absorbs the normal forces. This is why a slightly greater deformation can be observed in those regions than with the bending load case, but basically, there is less deformation (0.1337 mm) than with the grid infill (0.1613 mm).

**Figure 13:**
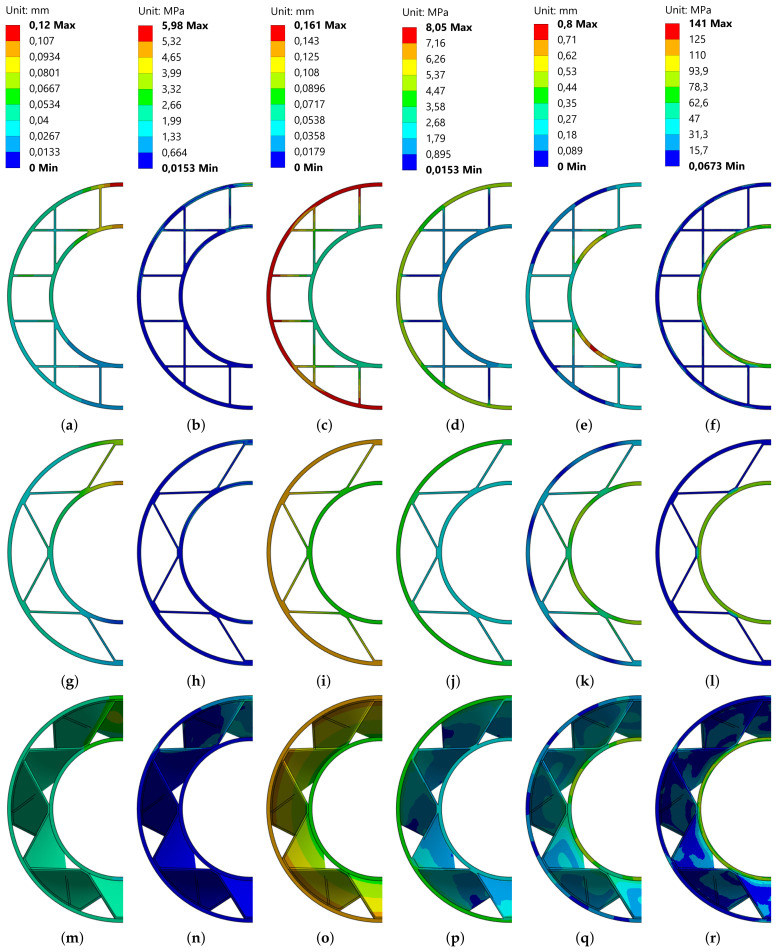
Analysis results (view in to x–z plane, see Figure 12): (**a**–**f**) Grid, (**g**–**l**) corrugated, (**m**–**r**) bi-corrugated, (**a**,**g**,**m**) bending deformation, (**b**,**h**,**n**) bending stress, (**c**,**i**,**o**) torsion deformation, (**d**,**j**,**p**) torsion stress, (**e**,**k**,**q**) pressure deformation, (**f**,**l**,**r**) pressure stress.

The bi-directional corrugated infill is arranged against the direction of rotation and should, therefore, be expected to have even better resistance behavior against twisting. The deformation of the normal corrugated infill is even less. The bi-directional infill also performs best under pure pressure load in load case 3. It can, therefore, be stated that an increase in stiffness could be achieved for at least the bending, torsion and internal pressure load cases with the same material consumption. Due to the directly proportional dependency of print time and material consumption, the print time will decrease further if the material usage reduces. Figure 13 shows the individual deformations and stress distributions of the different cross-sections.

## 4. Discussion

**Figure 14:**
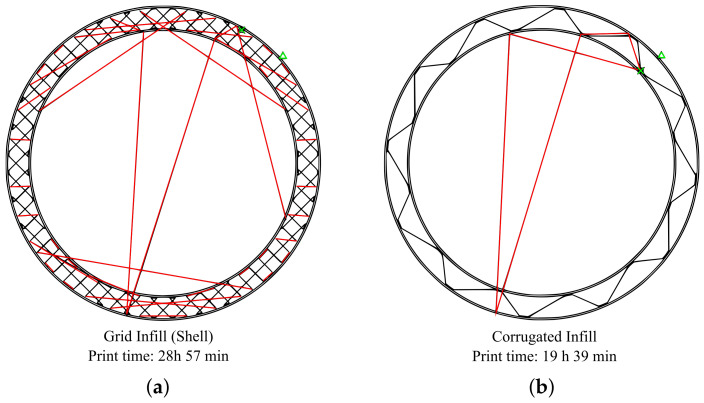
Print time example of two different infill types. Extrusion path (black lines); travel path (red lines); start and finish point (green mark). The underlying geometry is the test geometry from Figure 7, scaled with a scaling factor of 10: (**a**) Conventional grid infill; (**b**) Corrugated infill.

The results of the test series confirm a significant potential for the reduction in material consumption and print time by incorporating a hollowing operation into the slicing process. Further improvements are achieved when the generic grid infill is replaced with the novel corrugated infill structures that are optimized for hollow part geometry as described in Section 2.3. The optimization of the corrugated infill structure with regards to the printing process has also been verified. A comparison of G-codes of the same layer with grid infill versus corrugated infill (see Figure 14) shows that the number of individual extrusions and positioning moves (red) required to produce the infill is drastically reduced as the corrugated infill structure is printed in one continuous extrusion.

While the benefits of the novel infill structure are apparent, practical application is only possible under the consideration of certain criteria regarding part geometry. As the corrugated infill structure is defined by the number of contacting interfaces around the circumference, the infills density is proportional to the circumference of the part. As a result, application to parts with large variations in circumference can be impractical because some areas receive either too little or too much support. Furthermore, the introduction of a cavity into the part requires additional attention regarding printability, as overhangs may occur within the cavity that hinder a successful print. A method was implemented and tested to fill the volume in the upper part of the cavity so that overhangs do not exceed a given threshold. The results of the three-point bending test verify the issue of infill orientation for traditional infills, such as grid infill discussed in Section 2. The bending stiffness of the grid infill varies by a factor of three depending on its orientation relative to the parts outer surface. In a hollowed part, this results in a strong variation in bending stiffness along the parts circumference. Additionally, the infill shows anisotropic behavior when comparing a longitudinal and transversal core orientation. The corrugated infill structure introduced in this paper addresses the problem by generating the infill geometry based on the wall geometry, resulting in a uniform orientation throughout the circumference. The bending stiffnesses in the tests are found to be similar to that of the grid infill in its preferable orientation, including the anisotropic properties. The bi-directional variant of the corrugated infill improves on its bending stiffness and achieves a near isotropic bending response. The double-corrugated infill shows no difference between longitudinal and transversal loading and the lowest deformation of all specimens.

Overall, the method presented in this work offers several significant advantages over conventional approaches found in the literature. The innovative method presents a corrugated core structure, enhancing stiffness and optimizing material distribution, particularly for components subjected to bending stresses. Integrated directly into the slicing process, this method allows for an infill that adapts to the outer contours of the part, providing consistent support throughout the structure. The primary advantage of this new methodology compared to conventional infill patterns lies in the substantial reduction in material consumption and print time. Simulations and experimental results demonstrate that the new method can achieve up to 77% material savings and reduce printing time by up to 78% while maintaining comparable stiffness. These savings mark a significant advancement in additive manufacturing by not only reducing production costs but also promoting resource efficiency. Furthermore, this proposed infill pattern offers a more isotropic distribution of bending stiffness due to its geometry-aware design. Conventional infill patterns often exhibit anisotropic properties that can negatively impact the structural integrity of the final product. In contrast, the bi-directional and double-corrugated configurations of our infill pattern yield near-isotropic bending stiffness, leading to more uniform load distribution across the entire component. This represents a clear advantage over traditional infill strategies, which do not consider the specific geometry of the part in the infill pattern and can, therefore, result in inadequate stability in critical areas. In summary, the proposed algorithm provides a practical, slicer-integrated solution for enhancing stiffness, material efficiency and production time of FFF (fused filament fabrication) parts. Additionally, it is more user-friendly than alternative topology optimization methods, which often rely on finite element analysis and require greater expertise.

The principles of this corrugated infill methodology also hold promise for other additive manufacturing processes, such as selective laser sintering (SLS) and digital light processing (DLP). Both SLS and DLP processes can benefit from the improved structural integrity and reduced material consumption that come with a geometry-aware, corrugated infill. For SLS, which sinters powdered material layer-by-layer, a corrugated structure could optimize powder distribution and sintering paths, leading to enhanced material efficiency and potentially reducing post-processing needs. In DLP, where liquid resin is selectively cured, implementing a corrugated pattern could decrease resin consumption and curing time while still achieving similar or superior mechanical properties. Although each process may require specific adaptations to the infill design and slicing software, the fundamental benefits observed in FFF could be similarly achieved, making this approach broadly applicable and impactful across a variety of additive manufacturing technologies.

## 5. Conclusions

This study introduced a novel, geometry-aware infill pattern inspired by corrugated sandwich structures, specifically aimed at enhancing material efficiency, print speed and structural stiffness in fused filament fabrication (FFF) parts. The proposed method integrates directly into a slicer software, enabling continuous extrusion paths. The following key findings were identified during the investigation of the method:

- Material Savings: The hollowed structure combined with the corrugated infill achieves up to 77% reduction in material use, making it an effective approach for resource conservation.
- Print Time Reduction: By minimizing travel paths and implementing continuous extrusion, print times are decreased by up to 78%, optimizing production efficiency.
- Isotropic Stiffness: Unlike conventional infills, which often exhibit anisotropic mechanical properties, the corrugated design provides more uniform bending stiffness. In particular, the bi-directional variant demonstrated near-isotropic load distribution, enhancing reliability under diverse loading conditions.
- Ease of Integration: This infill strategy avoids the complexity of finite element analysis (FEA)-based optimizations, making it accessible for practical applications across a range of FFF projects.

While the proposed infill method shows promising results, certain limitations exist. The current algorithm requires hollowed parts with a consistent inner and outer contour per layer, making it less suitable for complex geometries with significant cross-sectional variation. Additionally, overhangs in the hollowed cavity may require added support to ensure print quality, and the infill density can vary with changing part dimensions. Future work should focus on extending this methodology to accommodate varying part geometries by introducing adaptive thresholds for infill density. Further development could also explore integration with other support structures or advanced shell-thickness adjustments for load-specific tuning. Incorporating this algorithm into popular open-source slicer software would enhance usability and accessibility, allowing a broader range of applications in the additive manufacturing community. In addition, the improvements found through the simulations should also be validated with the help of experiments. A testing machine has already been procured for this purpose, which achieves the appropriate resolution in the small load range.

## Figures and Tables

**Table 1:** The parameters of the test part geometries. The interface count and size were determined in such a way that the final component volume corresponds to the grid and grid shell infill.

Infill Type	Shell Thickness	Infill Settings
Grid	No pre-processing	Infill density: 25%
Grid (Shell)	8 mm	Infill density: 25%
Corrugated	8 mm	Interface count = 24 Interface size = 0.01 mm
Double- Corrugated	8 mm	Interface count = 24 Interface size = 0.01 mm

**Table 2:** Analysis data for different loadcases (Load case 1: Bending; Load case 2: Torsion; Load case 3: Pressure) of the pipe example. The first 3 rows are absolute values and the last 3 rows are ratio values of the infill types in relation to the grid infill (green cells are highlighted to show the best alternative to grid infill).

	Absolute Data					
	Load Case 1: Bending		Load Case 2: Torsion		Load Case 3: Pressure	
**Infill type**	$u_{1,max}$	$\sigma_{1,max}$	$u_{2,max}$	$\sigma_{2,max}$	$u_{3,max}$	$\sigma_{3,max}$
Grid	0.1201 mm	5.9797 MPa	0.1613 mm	8.0508 MPa	0.7966 mm	140.90 MPa
Corrugated	0.0951 mm	4.3432 MPa	0.1337 mm	5.1833 MPa	0.5780 mm	127.61 MPa
Bi-directional-Corrugated	0.0914 mm	4.5627 MPa	0.1384 mm	5.2617 MPa	0.5606 mm	123.88 MPa
	Relative values related to grid infill					
Grid	100%	100%	100%	100%	100%	100%
Corrugated	79%	73%	83%	64%	73%	91%
Bi-directional-Corrugated	76%	76%	86%	65%	70%	88%

## Data Availability

The original contributions presented in the study are included in the article, further inquiries can be directed to the corresponding author.
